# Congenital central nervous system anomalies: Ten-year single center experience on a challenging issue in perinatal medicine

**DOI:** 10.4274/jtgga.galenos.2018.2018.0079

**Published:** 2019-08-28

**Authors:** Emine Aydın, Atakan Tanacan, Melek Büyükeren, Hasan Uçkan, Murat Yurdakök, Mehmet Sinan Beksaç

**Affiliations:** 1 Clinic of Obstetrics and Gynecology, Kayseri Training and Research Hospital, Kayseri, Turkey; 2 Department of Obstetrics and Gynecology, Hacettepe University Faculty of Medicine, Ankara, Turkey; 3 Department of Child Health and Diseases, Neonatology Unit, Hacettepe University Faculty of Medicine, Ankara, Turkey

**Keywords:** Assessment, central nervous system, congenital abnormalities, neurosurgical procedure, prenatal diagnosis

## Abstract

**Objective:**

Our goal was to highlight the prenatal diagnosis and management of central nervous system (CNS) anomalies through sharing our clinic’s experience.

**Material and Methods:**

We evaluated prenatal findings and postnatal outcomes of neonates who had a CNS anomaly diagnosis in our clinic over a ten-year period. A total of 183 cases with various CNS anomalies were included in the study. Birth or termination preferences of mothers were recorded in all cases, and postnatal diagnosis concordance and prognosis after surgical procedures were evaluated in mothers who chose to continue the pregnancy.

**Results:**

The mean maternal age was 28.2±5.5 years, mean gravida was 2.2±1.3, and the mean gestational age at diagnosis was 30.5±5.5 weeks. Seventy-five out of 183 (41%) patients chose to terminate their pregnancy. Twenty babies (26.6%) in the termination of pregnancy group had additional anomalies. One hundred eight patients gave birth at our institution. The mean birth weight was 3060±647.5 g, the mean gestational week at delivery was 37.9±1.7 weeks, and mean APGAR score (5th minute) was 8.8±2.3. Four neonates died on the postpartum first day. The postnatal diagnosis of 60 of the 108 (55.5%) patients who gave birth was concordant with the prenatal diagnosis, and 32 of the 108 (29.6%) babies underwent surgical interventions.

**Conclusion:**

CNS anomalies have a broad spectrum and variable prognoses. This study highlights the limitations of prenatal diagnoses, and the need for parents to have this information in order to determine the course of their pregnancy and prepare themselves for the postnatal challenging treatment/rehabilitation process.

## Introduction

Central nervous system (CNS) anomalies are the second most common type of congenital defects after cardiac anomalies (1). Although CNS defects vary based on society and geography, they are reported to occur in 1 to 10 of every 1000 live births (2). Currently, this congenital defect group can be screened by measuring maternal serum alpha-fetoprotein (ms-AFP) levels. Furthermore, prenatal diagnosis is possible using ultrasonography (US) and/or fetal magnetic resonance imaging (MRI) (3,4). When these pregnancies result in birth, neonates with severe CNS anomalies require long-term intensive care, surgical intervention, and a prolonged treatment and rehabilitation process, all of which place a substantial material and spiritual burden on the families (2). Besides birth, these pregnancies can be terminated. Indeed, some parents with fetuses that exhibit anomalies during the prenatal period choose to terminate their pregnancy given the poor prognosis. On the other hand, some anomalies such as mild ventriculomegaly typically have a favorable outcome and physicians may choose conservative management options in such cases. Thus, antenatal counseling may be challenging both for families and physicians. For these reasons, CNS anomalies have attracted the attention of many researchers. Several studies have investigated the etiology of various conditions associated with such anomalies, sought to refine prenatal diagnostic methods, pursued alternatives to treatment and prevention, and have presented long-term follow-up data from infants affected by these anomalies (3,4).

In this study, we characterized the outcomes of pregnancies with fetal CNS anomalies in the prenatal period in our clinic.

## Material and Methods

This study consisted of 250 prenatally diagnosed CNS abnormalities between 2006 and 2016. The Hacettepe University Perinatology database was used for data collection. We evaluated the maternal age, obstetric history, gestational age at prenatal diagnosis, US findings (CNS anomaly type), karyotyping results (if performed), presence of additional anomalies other than those of the CNS, fetal MRI (if performed), pregnancy outcomes, perinatal complications, newborn information (gestational age at delivery, neonatal birth weight, APGAR scores), postpartum examination results (for the confirmation of prenatal diagnosis), postpartum surgical intervention (if performed), results of additional examinations in the neonatal intensive care unit (cranial US, MRI), prognosis of those born in our center, and long-term follow-up of accessible cases. Sixty-seven patients were excluded from the study due to missing data. Patients who were referred from other medical institutions who had been delivered at other hospitals were excluded from the study due to a lack of sufficient data, together with some of our own patients who had missing data (n=67).

In cases where parents chose to terminate their pregnancy, we analyzed the gestational age at the time of termination, types of anomalies, and results of the autopsy of the fetuses (in cases with parental permission). Statistical analyses were performed using the Statistical Package for the Social Sciences (SPSS.22^®^, IBM SPSS Statistics for Windows, Version 22.0. Armonk, NY: IBM Corp.) software package. The Kolmogorov-Smirnov test was used to evaluate the normal distribution of the data. Normally distributed data are presented as mean and standard deviations, whereas non-parametric data are presented as median (minimum-maximum values). The study protocol was approved by Hacettepe University Ethics Committee (GO 17/161). Written informed consents were obtained from all of the participants of the study.

## Results

Abnormalities (n=250) were classified as follows: anencephaly (n=4), neural tube defect (NTD)/Arnold-Chiari malformation (n=58), holoprosencephaly (HPE) (n=10), disorders of the corpus callosum (CC) (n=31), Dandy-Walker malformation (DWM) (n=30), mega cisterna magna (MCM) (n=13), vermian hypoplasia (n=2), porencephalic cysts (n=6), lissencephaly (n=3), hydranencephaly (n=21), craniosynostosis (n=4), mild (10 to 12 mm) ventriculomegaly (n=49), moderate (13 to 15 mm) ventriculomegaly (n=7), and severe (≥16 mm) ventriculomegaly (n=12).

After the exclusion of patients with missing data (n=67), the remaining cases (n=183) were as follows: anencephaly (n=4), NTD/Arnold-Chiari malformation (n=44), holoprosencephaly (n=7), disorders of the CC (n=25), DWM (n=20), MCM (n=5), vermian hypoplasia (n=2), porencephalic cysts (n=5), lissencephaly (n=2), hydranencephaly (n=20), craniosynostosis (n=3), mild (10 to 12 mm) ventriculomegaly (n=32), moderate (13 to 15 mm) ventriculomegaly (n=6), and severe (≥16 mm) ventriculomegaly (n=8).

The mean maternal age was 28.2±5.5 years, mean gravida was 2.2±1.3, and the mean gestational age at diagnosis was 30.5±5.5 weeks.

A total of 75 out of 183 mothers (41%) chose pregnancy termination. The remaining 108 mothers’ pregnancy follow-up and deliveries were performed at our center. The distribution of CNS anomalies in the termination group and the remaining patients are shown in Table 1.

### Termination group (n=75)

In the termination group, the mean maternal age was 27.9±5.4 years, mean gravida was 2.2±1.5, and the mean gestational age during prenatal diagnosis was 21.3±4.1 weeks. The distribution of anomalies in this group is shown in Table 1. Twenty babies (20/75, 26.6%) in this group had additional anomalies other than those of the CNS (Table 2).

Fetal MRI was performed on 5 patients in this group. Preliminary prenatal diagnoses were hydranencephaly (n=3), cerebellar hypoplasia (n=1), and CC agenesis (ACC) (n=1). ACC was additionally detected using MRI in all of the 3 patients with hydranencephaly. The preliminary diagnoses of ACC and cerebellar hypoplasia were consistent with fetal MRI results.

Mothers of 11 patients (11/75, 14.6%) agreed to a karyotype analysis. Nine of these eleven patients had normal karyotype results. In the two other cases, a triploidy and 46,XY,ins(12;2) case were detected. The 46,XY,ins(12;2) case had additional anomalies as indicated in Table 2. No additional anomalies were observed in the case of the triploidy.

### Patients who gave birth at our hospital (n=108)

The mean maternal age was 28.1±5.6 years, mean gravida was 2.1±1.3, and the mean gestational age at diagnosis was 29.2±5.1 weeks in this group. The distribution of anomalies in this group is shown in Table 1. Eight babies (8/108, 7.4%) in this group had additional anomalies other than those of the CNS (Table 3).

Fetal MRI was performed on 8 patients in this group. The preliminary prenatal diagnosis included mild ventriculomegaly (10-12 mm) (n=4), DWM (n=1), porencephalic cysts (n=1), ACC (n=1), and NTD/Arnold-Chiari malformation (n=1). The preliminary diagnosis of 6 cases was consistent with the fetal MRI result. The MRI diagnosis was consistent with ACC in the patient with pre-existing porencephalic cysts. Delay in brain sulcation was detected on MRI in the case of pre-diagnosis of ACC.

Mothers of 11 patients (11/75, 9.2%) agreed to a karyotype analysis. Nine of eleven patients had normal karyotype analysis results. Trisomy 18 was detected in one fetus and trisomy 13 was detected in another. These 2 fetuses’ additional anomalies are defined in Table 3.

### Postnatal outcomes of neonates (n=108)

The mean birth weight was 3060±647.5 g, mean gestational week at delivery was 37.9±1.7, and mean APGAR score (5^th^ minute) was 8.8±2.3. There were seven (6.5%) in vitro fertilization (IVF) pregnancies, the others were spontaneous pregnancies (93.5%). Sixty-nine of the neonates were male (63.8%) and 39 (36.2%) were female.

Intrauterine growth restriction (IUGR) was present in 12 neonates (11.1%). The remaining 96 neonates’ birth weights were compatible with the gestational week at delivery (88.9%). Patients with IUGR (n=12), had mild ventriculomegaly (n=3), severe ventriculomegaly (n=1), HPE (n=2), CC disorders (n=2), DWM (n=2), hydranencephaly (n=1), and NTD/Arnold-Chiari malformation (n=1).

Four neonates died in the neonatal intensive care unit on the postpartum first day. Three of these four babies had IUGR (1 DWM, 1 CC disorders, and 1 HPE). These three babies were born at term (39^th^, 40^th^, and 37^th^ gestational week, respectively). The remaining one neonate, with anencephaly, was born at the 33^rd^ gestational week and without IUGR. This fetus was referred to our center from another health care center with preterm prelabor rupture of the membranes. The remaining 104 neonates all underwent postnatal testing and treatment (if any) in the neonatology department.

### Neonates whose prenatal diagnosis was consistent with postnatal definitive diagnosis (n=60, 55.6%)

Postnatal examinations and imaging (US and/or MRI) were used for the definitive diagnosis of the CNS anomalies of the 108 neonates who were born at our center. Patients with a postnatal final diagnosis of anencephaly (n=1), NTD/Arnold-Chiari malformation (n=22), holoprosencephaly (n=3), DWM (n=6), vermian hypoplasia (n=1), hydranencephaly (n=3), craniosynostosis (n=1), and severe (≥16 mm) ventriculomegaly (n=5), were consistent with their prenatal diagnosis. Furthermore, diagnoses were consistent with the prenatal diagnosis of porencephalic cyst in one case, men who have sex with men (MSM) in one case, and CC disorders in 16 cases.

### Neonates whose prenatal diagnosis was discordant with postnatal definitive diagnosis (n=48, 45.4%)

In the porencephalic cyst group (n=4), ACC was detected in 3, and resorbed hematoma was detected in 1 neonate. In the MCM group (n=5), 4 were normal at the neonatal period. In the CC disorders group, 2 were normal at the neonatal period. In the moderate (13 to 15 mm) ventriculomegaly group (n=6), five fetuses were normal and one fetus had mild ventriculomegaly at the neonatal period. All patients in the mild (10 to 12 mm) ventriculomegaly group (n=32) had normal postnatal diagnostic results. In the postnatal period evaluation, 44 babies were totally normal.

### Surgical intervention outcomes

**Operated group:** Neurosurgical procedures (n=28, 26%) included the repair of myelomeningocele and ventriculo-peritoneal (VP) shunt insertions. These 28 neonates had NTD/Arnold-Chiari malformation (n=20), and patients with CC disorders (n=4), severe ventriculomegaly (n=1), hydranencephaly (n=1), cerebellar hypoplasia (n=1), and DWM (n=1) had undergone VP shunt operation due to hydrocephalus. One of these patients died at the postoperative 6^th^ month, the others survived.

Eleven neonates from this operated group received long-term follow-ups at our centers in the Children’s Hospital (Hacettepe Children’s Hospital). All 11 children had severe motor mental retardation (MMR). In two patients, neurogenic bladder was diagnosed. It was learned that the families of the other two NTD/Arnold-Chiari malformation patients, who could not be operated on, refused treatment and left the hospital with the neonates.

**Non-operated group:** There were 76 neonates (70%) that did not undergo surgery in our center. It was learned that 12 of the remaining 76 neonates who did not undergo surgery had died. The distribution of these 12 fetuses was as follows: anencephaly (n=1), mild ventriculomegaly (death due to kidney failure in the postnatal period) (n=1), moderate ventriculomegaly (postnatal diagnosis of Walker-Warburg syndrome) (n=1), HPE (n=2), ACC (n=1), DWM (n=2; one fetus had trisomy 18 and one fetus had trisomy 13), MSM (due to heart failure at the postnatal period) (n=1), porencephalic cyst (n=1), and hydranencephaly (n=1). One child who had a porencephalic cyst diagnosis prenatally and periventricular hemorrhage diagnosis in the postnatal period died at the age of six years.

A total of 31 of the remaining 64 un-operated patients continued their long-term follow-ups at our hospital. We could not track the information of the remaining 33 patients because they completed long-term follow-ups at other centers. Twenty-two of these 31 patients were found to be neurologically normal; these included cases of CC disorder (n=3) for which the postnatal diagnoses was CC hypoplasia, MSM (n=1), moderate ventriculomegaly (n=2), and mild ventriculomegaly (n=16).

The remaining nine children had the following postnatal diagnoses: epilepsy [n=4; prenatal diagnoses were mild ventriculomegaly (n=1), severe ventriculomegaly (n=1), and ACC (n=2)], metabolic disorder (n=1; methylmalonic acidemia, prenatal diagnosis was moderate ventriculomegaly), lalopathy (n=1; prenatal diagnosis was severe ventriculomegaly), and MMR [n=3; prenatal diagnoses were porencephalic cyst (n=1), CC disorders (n=1), and severe ventriculomegaly (n=1)]. One of the patients with MMR (the patient with CC disorder) had the diagnosis of trisomy 9 and monosomy 21 by postnatal genetic counseling. This child's family had not accepted a karyotype analysis in the prenatal period.

## Discussion

CNS malformations are the second most common cause of congenital anomalies, after congenital heart disease (5,6). Management and correct diagnosis remain a challenge for physicians. Many studies have been conducted to identify and classify major CNS anomalies. CNS malformations can be briefly classified as follows: NTD/Arnold-Chiari malformations (exencephaly, anencephaly, cephalocele, iniencephaly, spinal dysraphism/spina bifida, Arnold-Chiari type ll malformation), ventriculomegalies (mild, moderate, or severe), and those other than neural tube defects and ventriculomegaly [holoprosencephaly, CC disorders, cavum septi pellucidi, cavum vergae and cavum veli interpositi anomalies, posterior fossa abnormalities (DWM, MSM, Blake's pouch cyst, vermian hypoplasia), arachnoid cysts, aneurysm of the vein of Galen, schizencephaly, porencephalic cysts, hydranencephaly, lissencephaly, pachygyria, microgyria, heterotopias, and tumors] (1).

The screening and diagnostic process of these conditions started with ms-AFP screening, continued with USG, and now extends to fetal MRI. A thorough understanding of the normal sonographic appearance of the CNS across gestation is crucial for an accurate diagnosis because the presence or absence of a structure may be normal or abnormal depending on the age of the fetus. Poor timing of the examination, rather than poor sensitivity, can be an important factor in failing to detect a CNS abnormality (7). For example, a sonogram of the fetal brain at 14 weeks of gestation cannot detect ACC because the CC does not become sonographically apparent until 18 to 20 weeks of gestation and does not reach its final form until 28 to 30 weeks. Ideally, pregnancies at increased risk of fetal CNS anomalies and those with suspicious findings on a basic examination should undergo fetal neurosonography performed by physicians with expertise in this area. Our mean gestational age at diagnosis was 29.18±5.05 weeks. Late-diagnosed cases arise because patients live beyond reach of a healthcare center providing routine second-trimester screening.

MRI is an option for further evaluation in cases of diagnostic uncertainty when additional information will influence subsequent management of the pregnancy (7). The absence of shadowing artifacts and the better contrast resolution provided by fetal MRI compared with ultrasound makes it particularly suited for detailed imaging of the fetal brain (8,9). Fetal MRI is a relatively new method in our center, and our radiology team is more experienced at CNS malformations from congenital anomalies. There were 13 fetal MRIs in our series. Their US diagnosis, MRI diagnosis, and additional MRI findings are shown in Table 4. US diagnoses were correct in these patients and the MRIs gave additional findings in five patients.

Different anomalies and chromosomal and non-chromosomal syndromes can be accompanied by CNS anomaly subgroups (10,11,12). Their frequency and prognostic effects differ according to the anomalies (13). We identified 28 fetuses with extra structural abnormalities outside the CNS in our series (out of the total number of patients, including those in both termination and delivery groups). There were four cases of chromosomal abnormalities; trisomy 13, trisomy 18,46,XY,ins(12:2), and trisomy 9+monosomy 21 (postnatal diagnosis; prenatal diagnosis was unavailable because of lack of family consent). We also had two cases of Walker-Warburg syndrome and methylmalonic acidemia in the postnatal period.

When we evaluated perinatal, obstetric, and neonatal outcomes, we did not detect a greater frequency of IUGR, preterm delivery, or IVF pregnancies, contrary to previous work (14). Indeed, there is no clear evidence in literature for these associations with CNS anomalies.

Prenatal diagnosis is very important for parents deciding whether to continue or terminate their pregnancy, and to prepare themselves for the results. A recent review reported that the prenatal diagnosis of CNS anomalies and autopsy outcomes were 79.4% compatible (15). Also, it was reported that the prenatal diagnosis of CNS anomalies is the most consistent anomaly group in autopsy (15). In our series, prenatal and postnatal diagnoses were consistent in 55.6% of the diagnoses. On the other hand, the discordant group consisted of cases with prenatal diagnosis of porencephalic cyst, MCM, CC and moderate/mild ventriculomegaly. Porencephalic cysts are observed as a fluid-filled cavity in the cerebral hemisphere and they can involve the infratentorial or supratentorial space or both. The differential diagnosis can be challenging as tumoral lesions, arachnoid cysts and intracranial hemorrhagic changes may mimic the US findings according to a recent study in France (16). ACC was detected in 3, and resorbed hematoma was detected in 1 neonate whose prenatal diagnosis was porencephalic cyst in our study. MCM refers to enlargement of the cicterna magna to >10 mm on an oblique transverse plane with normal cerebellar hemispheres and vermis. In a systemic review of isolated prenatal posterior fossa malformations, the rates of additional CNS and other system anomalies were found as 12.6% and 16.6%, respectively (17). Furthermore, isolated MCM has a favorable outcome (18). The differential diagnosis of posterior fossa enlargement is another challenging subject in prenatal diagnosis and MCM, Blake’s pouch cyst and vermian hypoplasia may all cause similar US findings (19). MCM may resolve after delivery or it may be variant of normal anatomy (19). Four out of five neonates in this group had normal findings in the postnatal period.

Prenatal diagnosis for the disorders of the CC may be difficult for physicians. Developmental abnormalities of the CC include complete agenesis, partial agenesis, hypoplasia or hyperplasia. In a retrospective study that included 1722 prenatal US examinations, a positive predictive value of 47% (95% CI: 38-56) and a negative predictive value of 97% (95% CI: 96-98) were found for detecting agenesis of CC (20). In the CC disorders group, 2 were normal at the neonatal period in our study. Fetuses with isolated mild ventriculomegaly had a normal postnatal evaluation in more than 90% of cases and isolated moderate ventriculomegaly was associated with normal neonatal outcomes in 75% to 93% of cases, according to a recent review (21). In the moderate (13 to 15 mm) ventriculomegaly group (n=6), five fetuses were normal and one fetus had mild ventriculomegaly in the neonatal period. Additionally, all patients in the mild (10 to 12 mm) ventriculomegaly group (n=32) had normal postnatal diagnostic results in our study. Thus, our results were consistent with the literature and most of the discussed US findings in the discordant prenatal diagnosis group were probably variants of normal anatomy. On the other hand, a vast majority of the congenital CNS anomalies that were associated with adverse neonatal outcomes were detected prenatally in our institution.

The greatest problem with these pregnancies is the care of children after birth, and the spiritual and monetary burden on the family as well as the State. We were able to reach long-term follow-ups of 42 children in our series (operated and un-operated). Many of these children had mild and moderate ventriculomegaly (n=18). It is well known that an isolated, mild-to-moderate ventriculomegaly is linked to an abnormal outcome in 10%-20% of children (14), whereas ventriculomegaly with associated anomalies, or as part of a more complex syndrome, is characterized by abnormal outcomes in up to 40%-50% of children (22). In our study, all of the cases that resulted normally in follow-up were isolated. We could not determine the postnatal diagnosis of infants who died in the postnatal period who received a diagnosis of mild ventriculomegaly in the prenatal period because of families’ refusal of additional tests or autopsy. However, it is suspected that these deaths were related to the syndrome. Another patient with a prenatal diagnosis of mild ventriculomegaly was diagnosed as having epilepsy in the postnatal period.

Severe ventriculomegaly has often shown to be associated with poor neurologic outcomes in continued pregnancies (23). Long-term follow-ups of the three patients with severe ventriculomegaly in our series were as follows: one had severe MMR, one was epileptic, and one had lalopathy. Patients with NTD/Arnold-Chiari malformation who continued with post-operative follow-ups had severe MMR (n=11). Approximately 75% of patients who undergo myelomeningocele repair in infancy survive into early adulthood (24,25). Long-term prognosis is dependent upon the following factors: myelomeningocele level (thoracic and high lumbar defects are associated with greater disability and a higher risk of mortality compared with sacral and lower lumbar defects), the severity of the Chiari II malformation (a greater degree of hindbrain herniation is associated with a worse prognosis), and presence or absence of hydrocephalus (hydrocephalus is associated with greater disability and a higher risk of mortality). In addition, many of the complications (e.g., shunt malfunction, tethered cord, scoliosis, hydromyelia, and seizures) may negatively impact long-term prognosis. All of these details that predicted the prognosis were not completely clear in our data. However, it has previously been reported that 73% of these patients were neurologically symptomatic at any level (26). From this point of view, the current results support these data. For example, 6 patients with CC disorders whose follow-ups are ongoing at our center, support the findings of variable outcomes of CC disorders (27). It was seen that three of these patients were neurologically normal, two were epileptic, and one had severe MMR (the patient with trisomy 9+monosomy 21).

In the long-term follow-up of a patient who was diagnosed as having a porencephalic cyst in the prenatal period, hydrocephalus developed during the postnatal period, and the child now has severe MMR. The last patient under long-term follow-up is the child with MCM. It has been previously reported that, when isolated, MCM has a favorable outcome in 92% to 100% of cases (28).

The main strengths of our study were the relatively high sample size, which reflected over ten years’ data and the presence of long-term neonatal outcomes in most cases. However, its retrospective design and single-center experience are the main limitations of our study.

In conclusion, CNS anomalies have a broad spectrum and, even within disorders, their prognosis varies greatly. Diagnosis in the prenatal period is important for families so they can prepare themselves for the postnatal challenging treatment/rehabilitation process and determine the course of the pregnancy. Finally, these type of case series are becoming more and more important in preparing defensive reports to medic-legal issues.

## Figures and Tables

**Table 1:**
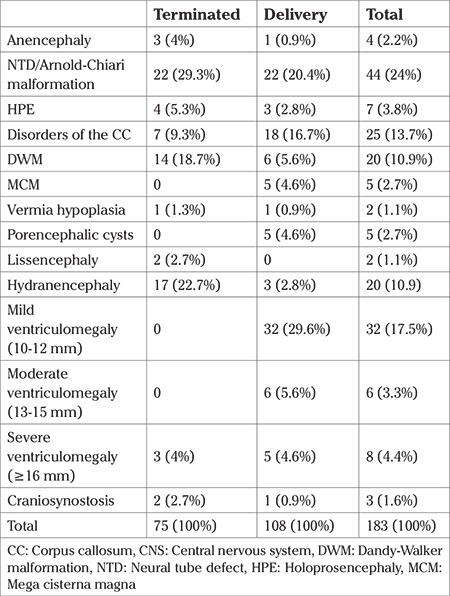
The distribution of CNS anomalies in the termination group

**Table 2:**
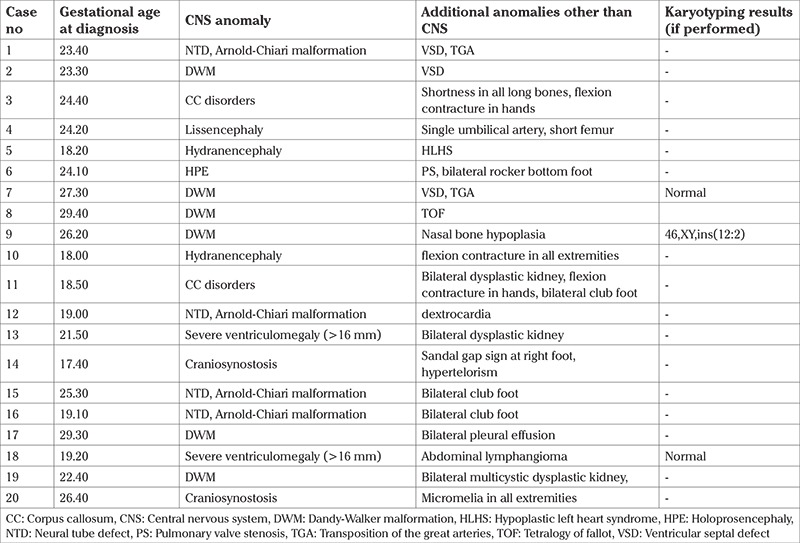
Additional anomalies in the termination group other than CNS

**Table 3:**
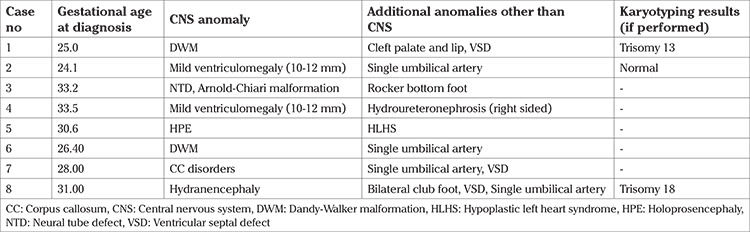
Additional anomalies in the delivery group other than CNS

**Table 4:**
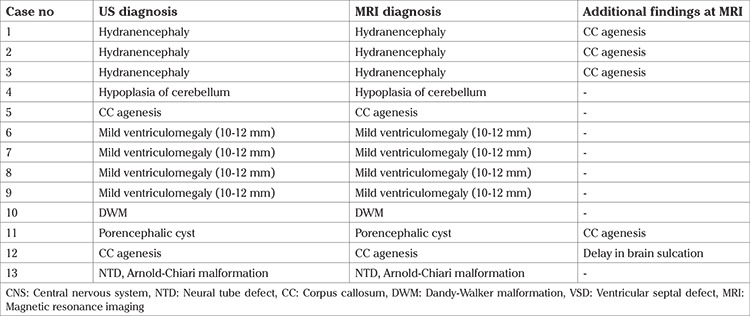
Additional findings in MRI
